# Health care experiences of people with Parkinson’s disease in Australia

**DOI:** 10.1186/s12877-023-04142-3

**Published:** 2023-07-12

**Authors:** Mary Danoudis, Sze-Ee Soh, Robert Iansek

**Affiliations:** 1 Clinical Research Centre for Movement Disorders and Gait, Kingston Centre, Parkinson’s Foundation Centre of Excellence, Monash Health, Cheltenham, VIC Australia; 2 Faculty of Medicine, Nursing and Health Sciences, School of Clinical Sciences, Monash University, Clayton, VIC Australia; 3 School of Public Health and Preventive Medicine, Monash University, 553 St Kilda Road, Melbourne, VIC Australia; 4 School of Primary and Allied Health Care, Monash University, Frankston, VIC Australia

**Keywords:** Parkinson’s disease, Parkinsonism, Health Care Experiences, Patient-centered care, Health Services

## Abstract

**Background:**

Little is known about the health care experiences of people with Parkinson’s disease (PwP) living in Australia. Exploring health care experiences can provide insight into service gaps which can then help direct quality improvement, such as improving communication between patients and health professionals.

**Methods:**

This study aimed to examine the health care experiences of a sample of PwP living in Australia using the Patient-Centered Questionnaire for Parkinson’s disease (PCQ-PD). Participants were recruited from four sources located in Victoria, Australia: (1) a metropolitan Movement Disorders Program (Group 1); (2) metropolitan based movement disorder neurologists working as sole practitioners and not within multidisciplinary teams (Group 2); (3) a regional based multidisciplinary PD program (Group 3); and (4) PD support groups in regional and rural Victorian towns without PD specialist programs (Group 4). Scores derived from the PCQ-PD included the overall patient-centered score (OPS), six sub-scale experience scores (SES) and the quality improvement scores (QIS). Health care experiences were compared between Groups 1, 2, 3 and 4 and multivariate linear regression models were used to explore factors contributing to patient-centeredness.

**Results:**

227 participants reported a mean (SD) OPS score of 1.8 (SD 0.5) with no significant differences between groups. The rating for the Tailored Information subscale was low, (mean 1.3, SD 0.5), with Group 2 having a significantly lower score, 1.1 (SD 0.5), compared to Group 1, 1.4 (SD 0.5) (*p* = 0.048). Experiences of Continuity of Care and Collaboration of Professionals were rated significantly lower by Group 2, 1.3 (SD 1.0) compared to Groups 1, 1.8 (SD 0.9) (*p* = 0.018) and 3, 2.1 (SD 0.8) (*p* = 0.002). Care aspects related to the Tailored Information subscale were prioritised for improvement by all groups. The main predictors of positive health care experiences were disease duration (coeff 0.02; 95% CI 0.00, 0.04) and living with another person (coeff 0.27: 95% CI 0.03, 0.51).

**Conclusion:**

This sample of participants with PD had poor experiences of several aspects of care known to be important in the provision of quality PD care. They prioritised the improvement of personalised health care information and better continuity of care and collaboration between health professionals.

**Supplementary Information:**

The online version contains supplementary material available at 10.1186/s12877-023-04142-3.

## Introduction

The importance of assessing the quality of health care by evaluating patient experiences has been recognised by health care organisations and government health departments for some time [1, 2]. Patient health care experiences are influenced by various aspects of health care delivery, such as access to appropriate services, communication with health care providers, provision of health information and involvement in care plans [3]. The importance of a positive health care experience for patients is well recognised with positive experiences being associated with better health care processes and outcomes [3]. Exploring health care experiences can provide insight into the quality of the care received and help determine if patient-centered care is being provided [3–5]. Information gained from patient experience data can inform health care providers on the gaps in their service provision. Identifying the gaps in care can direct health care quality improvements, such as improving communication between patients and health professionals, and help identify what initiatives to target, all of which are key to delivering high quality care [1, 6, 7].

The health care experiences for people with Parkinson’s disease (PwP) have been reported for those living in Europe, the Netherlands and North America [8–10] however little information exists for PwP living in Australia [11, 12]. Health care experiences reported for international populations cannot be readily generalised to those in Australia because of differences in factors such as models of care and health insurance coverage. The centres that participated in the North American study were PD centres of excellence that utilised a comprehensive care model advocated for PD [8]. In the Netherlands neurological clinics treating PwP are commonly integrated and networked [13] which is not a feature of Australia’s Parkinson’s health services. Coordinated multidisciplinary specialist PD teams are not universally available in Australia and are especially limited in regional areas [12]. These varying service models may influence the patient’s health care experiences differently. Additionally, differences have been shown to exist across a range of health care indicators for Australia compared to other OECD countries [14] such as all Australians having access to universal health insurance compared to only 37% of the North American population having government or social health insurance. These differences may result in differing access opportunities to health services between countries and subsequent differing health care experiences. If Australian health care is to meet the needs of PwP living in Australia, then organisations need to know how they are performing from the patient’s perspective. Understanding their experiences can provide health service organisations with information on specific areas needing improvement thereby improving the quality of care provided to PwP within the Australian context. Health care services can therefore be better prepared with strategies, such as the use of communication tools, that PwP find to be effective and as meeting their needs. It is important to therefore explore the Australian experience in greater detail.

Comparison between regional and urban Australian health care for PwP has been limited to differences in clinical management, patient satisfaction and utilisation of health care services [12]. The comparison of health care experiences of PwP living in regional centres to those living in metropolitan centres has also not been examined within the Australian context. Thus, the overall aim of this study was to describe the health care experiences of a sample of PwP living in Australia. Specific objectives were to:

- Compare the experiences of a sample of PwP living in Australia who receive care from multidisciplinary PD specialist services with those who receive care from sole practitioners providing usual care.
- Examine the experiences of a sample of PwP living in a metropolitan city compared to those living in regional towns.
- Identify the demographic and clinical factors that contribute to health care experiences of PwP living in Australia.

## Methods

This study was approved by the Monash Health Human Research Ethics Committee (HREC Reference number LNR/16/MonH/28). Informed consent was provided by participants when they marked the consent box provided in the de-identified questionnaire that stated “Yes I have read the information and I understand what I am being asked to do, and I consent to participate”. In addition, participants who completed and returned the questionnaire were deemed to have sufficient decisional capacity thereby sufficient capacity to provide informed consent for this non-interventional, no/low risk study. The methods used in this study were carried out in accordance with relevant guidelines and regulations and adhere to the tenets of the Declaration of Helsinki.

### Study design and participants

Purposive sampling was used to recruit participants from four sources: (1) a metropolitan comprehensive multidisciplinary Movement Disorders Program (MDP) based in the state of Victoria, Australia (Group 1); (2) specialist movement disorder neurologists based in metropolitan Victoria who worked as sole practitioners and not within specialist multidisciplinary PD programs (Group 2); (3) a multidisciplinary PD program located in a regional centre in Victoria, Australia (Group 3); and (4) Fight Parkinson’s (the peak body supporting PwP in Victoria) support groups located in regional and rural towns across the state where there were no specialist multidisciplinary PD programs (Group 4).

Participants were eligible for the study if they (1) had a diagnosis of idiopathic PD, or Parkinsonism, confirmed by a neurologist or medical practitioner; (2) had received treatment for their PD during the last 12 months; and (3) had sufficient English to complete the questionnaire. Adults of any age were eligible. Participants were screened for eligibility by the project coordinator during the recruitment process.

### Recruitment process

#### Group 1

Group 1 participants were identified from the MDP electronic database or by a MDP clinician when they attended the service in person. Data extracted from the database included the names and addresses of consecutive patients with a diagnosis of PD or Parkinsonism who had attended the MDP in the 12 months up to the commencement of the study. Administrative staff, or the study’s coordinator, mailed an explanatory statement and an invitation to participate to potential participants. Those interested were instructed to contact the coordinator who then screened their eligibility before sending them a copy of the questionnaire together with a pre-paid return envelope. Eligible participants were also identified and screened by their treating health professional when they attended the MDP in person. Potential participants were provided with an explanatory statement about the study by the clinician, who also supplied them with a copy of the questionnaire to complete at home if they were interested in participating and a pre-paid addressed envelope for return.

#### Group 2

Patients eligible for group 2 were recruited by two means. Firstly, fliers outlining the study were provided to participating PD specialist neurologists for distribution to their current patients with PD. The flier instructed interested participants to contact the study’s coordinator for additional information and to obtain a copy of the questionnaire. Those who made contact were screened for eligibility by the coordinator who sent a copy of the questionnaire along with a pre-paid return envelope to eligible participants. Secondly, participants with PD who were included in a prior study because they received health care from Melbourne based private neurologists working as sole practitioners, were identified from the study’s database. Potential participants were sent a letter explaining the study. Those interested in participating contacted the study’s coordinator, who determined if they were eligible before mailing them a copy of the questionnaire and a prepaid return envelope.

#### Group 3

Patients with PD who were current patients of a regional specialist PD program were informed about the study by the program’s neurologist. Those interested were asked to consent to their contact details being forwarded to the study’s coordinator. The coordinator contacted them, screened their eligibility and sent those eligible and interested in participating a copy of the questionnaire and a pre-paid return envelope.

#### Group 4

Fight Parkinson’s promoted this study to their regional and rural support groups. The support group leaders provided members with a flier that outlined the study. Those interested in participating contacted the coordinator who screened their eligibility before sending them a copy of the questionnaire along with the explanatory statement and a pre-paid return envelope.

### Outcomes

A self-administered questionnaire was used to capture data for the evaluation of patient health care experiences. The questionnaire included background questions such as sex, age, social supports and disease duration. To describe the PD characteristics of the sample, the following PD specific measures were also included. Participants were asked to self-report disease stage according to the Hoehn and Yahr staging scale [15]. Respondents also indicated the impact of PD on the motor aspects of their experiences of daily living using the validated MDS-Unified Parkinson’s disease Rating Scale (MDS-UPDRS) Part II [16]. The overall score was calculated by summing the scores for each of the 13 questions, with higher scores indicating greater impact of PD on function [16]. Health related quality of life (HRQOL) was measured using the Parkinson’s disease Questionnaire-39 (PDQ-39) [17]. The dimension scores were determined according to methods described by the developers of the tool [17]. The summary index score (SI) was calculated by summing the 8 dimension total scores then dividing by eight, yielding a possible score between 0 and 100 [17]. Higher scores of the SI reflected worse HRQOL. The validated 30 item Non-Motor Symptom Questionnaire (NMSQ) was used to measure the number of non- motor PD symptoms that participants experienced [18].

The primary outcome for this study was patient experience which was measured using the Patient-Centered Questionnaire for Parkinson’s disease (PCQ-PD) [9]. The PCQ-PD questions were based on key elements of patient-centeredness as defined by the Picker Institute [19] and the World Health Organization (WHO) [20]. THE PCQ-PD includes questions regarding the needs associated with in-patient and out-patient care as well as the disciplines typically providing care [9]. The scores calculated for the PCQ-PD included the overall patient-centeredness score (OPS, range 0–3), the six subscale experience scores (SES, range 0–3) and the quality improvement scores (QIS, range 0–9). Respondents rated their experiences on a range of aspects of care which were used to calculate the SES and OPS. Participants prioritised 44 aspects of health care and from their responses the QIS were determined. Calculations for all scores were undertaken according to the directions of the questionnaire developers [9].

The original version of the PCQ-PD was validated in a Netherland’s population and was found to have acceptable construct validity and appropriate internal consistency and reliability [9]. The Netherland’s version of the PCQ-PD was translated into English and cross-cultural validation indicated that the instrument had adequate validity with North American populations [8]. The English version of the PCQ-PD was used in this current study [9]. The questionnaire was reviewed by five PD specialist health professionals and three patients with PD to ensure the content was relevant to the Australian health care system and the language used was easily understood. Based on the feedback received, minor changes were made to the wording to improve clarity. The term “professional caregivers” was changed to “health professionals”, and questions that were not applicable to the Australian heath care setting, such as “have you been informed about the reimbursement of Parkinson medication” were removed. Questions relating to a neuropsychologist were also added to the questionnaire because this health professional is often involved in PD treatment in the Australian setting. Six questions in the original PCQ-PD that were only asked about the neurologist and nurse were added to the questions for the other health professionals due to the relevance to the Australian health care system. Health professionals included in the questionnaire were the neurologist, nurse, physiotherapist, occupational therapist, speech pathologist, social worker, neuropsychologist and the general practitioner.

### Analysis

Descriptive statistics were used to summarise the demographic and clinical characteristics and the health care experiences of participants.

Differences in health care experiences between groups were examined using ANOVA for continuous and ordinal data, and chi-squared test of goodness of fit for nominal data. The SPSS ‘exclude cases pairwise’ option was used to adjust for missing data. Post-hoc comparisons were performed with Bonferroni adjustment for multiple comparisons.

Two multivariate linear regression models were used to identify the demographic and clinical factors that contributed to the participants’ health care experiences. The dependent variable was the OPS derived from the PCQ-PD and the independent variables were participant demographics and clinical factors. The first model included receipt of care from different health care providers as the only predictor, whilst the second included factors such as age of onset of PD, number of comorbidities, disease severity according to Hoehn and Yahr stage and health related quality of life, to experiences of patient-centeredness all of which were entered simultaneously. Highly correlated variables were identified using the variance inflation factor (VIF) with values > 3 indicating the presence of multicollinearity. Model findings were reported as coefficients (coeff) with 95% confidence intervals (CI), and *p* < 0.05 was considered to be statistically significant.

## Results

### Demographic and clinical characteristics

Questionnaires were provided to 248 interested participants and returned by 229 (response rate 92%). Responses from two returned questionnaires were not included in the analysis because of ineligibility due to diagnosis of drug induced tremor and not completing the PCQ-PD and PD specific measures. The demographic and clinical characteristics of the 227 participants and for each of the four groups are reported in Table 1. Significant differences were found between groups for disease severity and functional disability as measured by the MDS-UPDRS Part II.

**Table 1:** Demographic and clinical characteristics of all participants and for the four groups

Characteristic	All participants (*n* = 227)	Group 1 (*n* = 82)	Group 2 (*n* = 38)	Group 3 (*n* = 39)	Group 4 (*n* = 68)
Sex, *n* (%)					
Male Female	138 (61)^!^ 88 (39)	46 (56) 36 (44)	25 (66) 13 (34)	28 (74)^!^ 10 (26)	39 (57) 29 (43)
Age at diagnosis, years	73.1 (8.3)^!^	74.5 (9.2)^!^	71.5 (8.6)^!^	73.7 (7.2)	72.0 (7.4)^!^
First language, *n* (%)					
English Not English	222 (98) ^!^ 4 (2)	78 (95) 4 (5)	37 (100)^!^ -	39 (100) -	68 (100) -
Education, *n* (%)					
< Year 11 Year 12 TAFE University	92 (41)^!^ 25 (11) 64 (28) 45 (20)	29 (35) 9 (11) 22 (27) 22 (27)	13 (34) 4 (11) 14 (37) 7 (18)	22 (56) 3 (8) 7 (18) 7 (18)	28 (42)^!^ 9 (13) 21 (31) 9 (13)
Living situation, *n* (%)					
Alone Not alone	36 (16) 191 (84)	15 (18) 67 (82)	8 (21) 30 (79)	5 (13) 34 (87)	8 (12) 60 (88)
Presence of carer, *n* (%)					
No carer Has carer	72 (32)^!^ 152 (68)	24 (30)^!^ 57 (70)	16 (43)^!^ 21 (57)	17 (44) 22 (56)	15 (22)^!^ 52 (78)
PD Diagnosis, *n* (%)					
Idiopathic PD Parkinsonism	215 (95) 12 (5)	76 (93) 6 (7)	36 (95) 2 (5)	38 (97) 1 (3)	65 (96) 3 (4)
PD duration, years	7.3 (5.4)^!^	7.5 (6.0)^!^	7.5 (4.5)	6.7 (5.3)	7.3 (5.4)^!^
HY stage	2.4 (1.0)^!^	2.6 (1.0)^!*^	2.2 (1.1)	2.0 (1.1)^!*#^	2.6 (0.9)^#^
MDS-UPDRS II (M-EDL)	15.4 (9.7)^!^	16.5(10.8)^!^	13.8 (9.5)^!^	11.5(7.6)^!^^	17.0(8.6)^!^^
PDQ-39 summary index	27.3 (16.0)^!^	28.1(17.0)^!^	23.5 (14.6)^!^	23.1(15.0)^!^	31.7 (15)^!^
NMSQ	11.6 (5.7)^!^	11.1 (5.9)^!^	10.4 (5.4)^!^	11.2 (5.4)^!^	12.9 (5.5)^!^

All data reported as mean (SD) unless stated otherwise. Valid percentage reported if data missing

^!^ Data missing

^*^Post-hoc comparisons with Bonferonni adjustment indicate *p* = 0.024;

^#^Post-hoc comparisons with Bonferroni adjustment indicate *p* = 0.042;

^^^Post-hoc comparisons with Bonferroni adjustment indicate *p* = 0.037

### Overall patient-centeredness scores and subscale experience scores (OPS and SES)

#### Health care experiences

Overall patient-centeredness scores and the six subscale experience scores are presented in Table 2. A lower SES indicates poorer health care experiences, with 0 being the most negative and 3 being the most positive experience. Experience of patient centered care was moderate with a mean OPS score of 1.8 (SD 0.5). Interpretation of the OPS by this current study was in keeping with the developers of the PCQ-PD who described their OPS of 1.69 (SD 0.45) as moderate [9]. The OPS did not differ significantly between the four groups. Inspection of the six subscale scores for all participants found their poorest experience was for the provision of tailored information. Post-hoc comparisons indicated that Group 2’s experience of provision of information, mean 1.1 (SD 0.5), was significantly poorer compared to that of Group 1, mean 1.4 (SD 0.5), (p = 0.048). Involvement in decision making and provision of emotional support were also rated poorly, with no significant differences between groups. Experiences of receiving continuity of care and collaboration of professionals were rated significantly poorer by Group 2, mean 1.3 (SD 1.0) compared to Group 1, mean 1.8 (SD 0.9) (*p* = 0.018) and Group 3, mean 2.1 (SD 0.8) (*p* = 0.002).

**Table 2:** Overall Patient-Centeredness Scores (OPS) and Subscale Experience Scores for each of the six subscales for all participants and for the four groups

	All participants *(n* = 227*)*	Group 1 (*n* = 82)	Group 2 (*n* = 38)	Group 3 (*n* = 39)	Group 4 (*n* = 68)
OPS	1.8 (0.5)	1.8 (0.5)	1.7 (0.6)	1.9 (0.5)	1.8 (0.6)
Subscale A *Involvement in decision making*	1.6 (0.6)	1.5 (0.6)	1.8 (0.7)	1.8 (0.6)	1.6 (0.7)
Subscale B *Provision of tailored information*	1.3 (0.5)	1.4 (0.5) ^*^	1.1 (0.5) ^*^	1.3 (0.6)	1.4 (0.5)
Subscale C *Accessibility of health care*	2.2 (0.6)	2.1 (0.7)	2.2 (0.8)	2.2 (0.6)	2.3 (0.5)
Subscale D *Empathy & PD expertise*	2.5 (0.5)	2.6 (0.4)	2.5 (0.6)	2.6 (0.4)	2.4 (0.6)
Subscale E *Continuity and collaboration of professionals*	1.7 (0.9)	1.8 (0.9) ^#^	1.3 (1.0) ^#^^	2.1 (0.8) ^	1.6 (0.9)
Subscale F *Emotional support*	1.6 (1.0)	1.6 (2.0)	1.4 (1.1)	1.8 (0.9)	1.5 (1.0)

All data reported as mean (SD)

Scores for OPS and Subscales range from 0 (most negative experience) to 3 (most positive experience)

^*^Post-hoc comparisons with Bonferonni adjustment indicate *p* = 0.048

^#^ Post-hoc comparisons with Bonferonni adjustment indicate p = 0.018

^^^Post-hoc comparisons with Bonferroni adjustment indicate *p* = 0.002

### Quality improvement scores (QIS)

#### Priorities for improvement

Quality improvement scores, ranging from 0 (low priority for improvement) to 9 (high priority for improvement), were inspected for all 44 priority items. The QIS, a continuous variable, was transformed into two categorical variables, high and low QIS, to simplify the clinical interpretation of the results, a method supported by prior studies [21]. The cut off score for high improvement priority was set at equal or greater than the mean of 4.5 (≥ 4.5). The items that were prioritised the highest for improvement were from the provision of tailored information subscale (Table 3). Being informed about what health professionals discussed with each other about the person’s treatment was rated as the item with the highest need for improvement overall. Inspection of the QIS for the individual groups also showed Group 2 had the greatest number of items with a high priority rating for improvement (Table 3).

**Table 3:** Quality Improvement Scores (QIS) reported for any of the 44 items with a high priority score. The cut off score for high improvement priority was set at equal or greater than the mean of 4.5 (≥ 4.5)

Care aspects with high priority for improvement	Subscale items connected to aspect of care	All participants (*n* = 227)	QIS			
			Group 1 (*n* = 82)	Group 2 (*n* = 38)	Group 3 (*n* = 39)	Group 4 (*n* = 68)
Informed about what health professionals discuss regarding treatment	Provision of tailored information	5.0 (2.9)	4.8 (2.7)	5.5 (3.1)	4.7 (3.0)	5.2 (3.1)
Information on alternative therapies	Provision of tailored information	4.5 (3.0)	4.3 (3.0)	5.1 (3.3)	4.5 (3.0)	4.4 (3.0)
Advanced treatment options	Provision of tailored information	4.4 (3.1)	4.5 (3.2)	4.6 (3.0)	4.6 (2.4)	4.1 (3.3)
Contact after starting PD med	Provision of tailored information	4.4 (3.2)	3.9 (3.0)	5.7 (3.0)	3.7 (3.5)	4.5 (3.1)
Know treatment options offered by different health professionals	Provision of tailored information	4.3 (2.3)	4.0 (1.7)	5.2 (2.6)	3.9 (2.5)	4.4 (2.5)
Health professionals support when dealing with personal relationship changes	Emotional support	4.3 (3.3)	3.4 (3.2)	5.2 (3.6)	3.6 (3.1)	5.3 (2.9)
Health professionals make mutual agreements for treatment options	Continuity of care	4.0 (3.0)	3.5 (2.9)	5.3 (2.7)	4.0 (3.1)	3.8 (3.0)
HP support when problems with employment due to PD	Emotional support	3.9 (3.6)	3.8 (3.8)	5.0 (3.7)	0 (0.0)	4.5 (3.3)
One person assigned to contact	Continuity of care	3.9 (3.6)	3.8 (3.5)	5.0 (3.5)	2.2 (3.5)	4.2 (3.5)
Ability to drive a car	Provision of tailored information	3.4 (3.2)	3.2 (3.2)	4.7 (3.4)	3.5 (3.2)	3.0 (3.0)
Differing physicians collaborate	Continuity of care	2.8 (3.4)	2.7 (3.2)	5.0 (3.7)	2.3 (4.5)	1.8 (2.8)
Health professionals provide support after diagnosis	Emotional support	2.8 (2.9)	2.2 (2.6)	4.7 (4.1)	2.0 (2.3)	3.1 (2.9)
Neurologist & PD nurse collaborate about treatment	Continuity of care	2.0 (3.0)	1.6 (2.9)	6.0 (5.2)	1.3 (2.4)	2.1 (2.8)

QIS reported as mean (SD)

Scores range from 0 (low priority for improvement) to 9 (high priority for improvement)

### Contribution of demographic and clinical factors and care received from different health care providers to overall patient-centeredness

#### Contributing factors to health care experiences

Associations between receipt of care from different health care providers and demographic and clinical factors such as disease duration, disease severity and HRQOL with overall patient-centeredness are detailed in Table 4. Receipt of care from different health care providers was not found to be a significant predictor of overall patient-centeredness as measured with the OPS score in the first model. The multivariate model showed that living with another person was significantly associated with overall patient-centeredness (Coeff 0.27, 95% CI 0.03 to 0.51). Duration of disease was also a significant predictor (Coeff 0.02, 95% CI 0.00 to 0.04) although this model only explained 1.6% of the overall variance in OPS scores.

**Table 4:** Multivariate linear regression models to examine the contribution of receipt of care from different health care providers and demographic and clinical factors to overall patient-centeredness

Variables	Overall patient-centeredness scores					
	Model 1 (univariate)			Model 2 (multivariate)		
	Coeff	95%CI	R^2^	Coeff	95%CI	R^2^
Group						
Metropolitan specialised MDP program Metropolitan non-specialised program Regional specialised program Regional non-specialised program	- -0.16 0.07 -0.09	Reference -0.37, 0.05 -0.14, 0.27 -0.26, 0.09	- 1.04 0.18 0.43	- -0.18 0.01 -0.12	Reference -0.43, 0.07 -0.25, 0.26 -0.33, 0.09	- 1.15 0.00 0.73
Age at diagnosis, years				-0.00	-0.01, 0.01	0.11
Sex						
Male Female				- 0.11	Reference -0.07, 0.29	- 0.77
Education						
< Year 11 Year 12 TAFE University				- -0.02 0.02 -0.06	Reference -0.31, 0.28 -0.19, 0.23 -0.29, 0.17	- 0.01 0.01 0.14
Living situation						
Alone Not alone				- **0.27**	Reference **0.03, 0.51**	- **2.90**
PD duration, years				**0.02**	**0.00, 0.04**	**2.22**
HY stage				-0.01	-0.11, 0.09	0.04
Overall HRQOL				-0.00	-0.01, 0.00	0.18

Coeff: Coefficient that reflects the degree of change in overall patient-centeredness scores for every one unit of change in the predictor variable; R^2^: unique contribution of each predictor variable to the total variance in overall patient-centeredness scores expressed as a percentage

## Discussion

This study provides information about the health care experiences of a sample of PwP living in Australia. Patient-centeredness was moderate overall with no significant differences between groups. Participants in this study gave a low rating to their experiences of the following aspects of care: receiving tailored health information; involvement in decision making; continuity of care and collaboration between clinicians; and emotional support. The provision of tailored information was given the highest priority for improvement by all participants. Care provided by specialist multidisciplinary services or sole practitioners in metropolitan or regional locations was not significantly associate with overall patient-centeredness. The significant predictors of overall patient-centeredness were living with another person and disease duration.

Patient-centeredness was moderate for this sample of PwP living in Australia, similar to findings from prior studies [8, 9]. It was surprising, however, that differences in the OPS were not observed between the four groups given that Groups 1 and 3 were multidisciplinary specialist PD centres. Such specialist PD services could be expected to be best suited to deliver patient-centered care [13, 22]. This non- significant finding may be related to features of our study design. Firstly, this study may have been underpowered to detect differences in patient-centeredness between groups. Additionally the PCQ-PD may lack sufficient discriminative power to identify actual differences between groups. Confounders not recorded by this study, such as barriers to accessing specialist PD health care services [23], and socioeconomic status may also have contributed to potential differences not being identified.

Having access to appropriate and relevant information is critical in the self-management of PD [13, 24, 25]. This current study found participants had poor experiences of receiving tailored information. Participants prioritised the improvement of provision of information on various aspects of care including what health professionals discussed between themselves regarding their treatment options and alternative therapies. Previous studies have shown that PwP vary greatly in what they perceive to be their worst symptoms and their health care preferences [8] which can impact on their preferences for how and what information is provided [26]. The diverse information requirements of patients require health care professionals to identify what information best meets the needs of each patient [27, 28]. Factors, such as age and cognitive function that may impact on a person’s capacity to make sense of information and to understand its relevance, and their ability to remember what they are told, also need to be considered [28, 29]. Whilst similar findings were reported by prior studies [8, 9, 29, 30] we did not explore the barriers to the provision of information in this study. Thus, further qualitative studies are needed to identify what the patient wants to know so that health care professionals can tailor the information to meet the individual’s needs while taking into consideration personal barriers [25].

Participants in this study rated their involvement in shared decision making with health care professionals to be poor. The benefits patients experience when they participate in clinical decision making are well documented and PwP have reported that they want to be involved in decision making [25, 31]. Barriers to PwP being involved in shared clinical decision making, as reported by prior studies, include a lack of knowledge on treatment options and when treatments should be commenced, their perception that they had no choice in the shared decision making process, the lack of time to discuss treatment options when seeing the doctor and not seeing the same health professional on repeat visits to discuss options [25]. This current study did not explore participation barriers however known barriers, such as not receiving information on treatment options and not having one key contact person, were given a high priority for improvement by participants. It is therefore important that health services ensure their care models are not inadvertently creating barriers to patient – clinician shared decision making processes.

Experiences of continuity of care and collaboration of professionals were rated low, similar to findings from prior studies [9, 29, 30]. Of note is the significantly poorer experience of this aspect of care by Group 2 compared to Groups 1 and 3 which provided PD specialist services, and Group 2’s high prioritisation for its improvement. Group 2 indicated that health care professionals needed to improve how they reached consensus on treatment plans, neurologists and nurses needed to collaborate better, physicians needed to collaborate better and they wanted to have one person assigned to them to act as the primary contact person. These findings can help direct health providers, in particular those who do not work within PD specialist multidisciplinary teams, on how best to improve their services in order to meet the needs of their patients.

The experience of receiving emotional support was also given a low rating by participants of this study. Emotional support from family, friends and health care professionals is important to help the PwP maintain a good QOL [32]. Psychosocial support is frequently provided by specialist PD nurses [33] and social workers or counsellors [34]. Participants in this current study indicated a low use of nursing (n = 81, 36%) and social work (n = 38, 17%) overall (Supplementary Table 1). Given that associations between experiencing emotional support and utilisation of these services were not examined, further studies are needed to investigate factors associated with emotional support experiences such as availability of suitably trained health care workers to discuss emotional issues [35].

The multivariate linear regression modelling demonstrated patient-centeredness was not significantly associated with the receipt of care from the different health care providers. In addition, there was no significant association between overall patient-centeredness and provision of care by health care providers in metropolitan and regional regions. The care provider and demographic and clinical factors accounted for less than 2% of the overall variance in overall patient-centeredness. This finding was in keeping with a prior study where differences in patient-centeredness between 20 PD centres of excellence accounted from between 1 and 6% of the variance [8]. Taking into consideration the complexity of PD, the variability of the experiences of the disorder between individuals and within an individual, it is not surprising that multiple personal factors may be associated with the person’s health care experiences [5, 7, 36]. Thus, there is a need for further research to identify what other confounding factors may contribute to the positive or negative experience of patient centred care. This information is needed to assist health care providers in the planning, development and evaluation of care that meets the needs of the individual.

Interpretation of the current study’s findings needs to be considered within the Australian context. The Australian Commission on Safety and Quality in Health Care (ACSQHC) has endorsed patient-centered care as an independent measure of the quality of health care services people receive [1]. Key descriptors of patient-centered care used by the ACSQHC correspond with the 6 domains of the PCQ-PD. The ACSQHC standards for patient centered care approaches require Australian health care services to partner with care recipients within a patient centered care approach [1]. Our findings suggest there remains a need for Australian health services providing care for PwP to review their processes to ensure they are meeting Australian Health Care standards.

Findings from this study can be used to support the involvement of PwP in quality improvement projects that aim to improve the health care services available to them. For example, the findings can inform the development of online resources for PwP and carers, regarding PD related health information as well as patient-centered communication tools that support greater involvement of PwP in the clinical decision making process.

### Limitations

A number of limitations need to be noted. Firstly, clinicians, one of whom is an author of this report, handed out questionnaires to eligible patients for Group 1. However, in order to avoid any sense of coercion and to ensure confidentiality and anonymity, participants did not return the questionnaire to the clinician. The clinician was also not involved in assisting participants with questionnaire completion. Whilst all participants from Group 3 were identified by their neurologist who is one of the study authors, he was not involved in the distribution or administration of the questionnaire. The neurologist also did not know which of his patients agreed to participate in the study. To further minimise selection bias, consecutive patients listed on the MDP (Group 1) data base and all patients registered with the regional PD program (Group 3) were invited to participate. We also acknowledge that given the design of this study and that questionnaires were only administered at one point in time, causality cannot be inferred. Nor are we able to draw firm conclusions on which model of care are more likely to improve the health care experiences of PwP.

The number of participants in this sample was small considering Victoria’s total PD population was approximately 57,000 people at the time the study was conducted as reported by Fight Parkinson’s. In addition, generalising the findings from this current study to the broader PD population in Victoria and Australia may be limited particularly as a result of our recruitment strategy. Further sampling of individuals, particularly those receiving care from major metropolitan and regional hospitals are needed to confirm and extend the generalisability of our findings. The findings, however, do provide new information relevant to Australia that suggest Australian health care providers may not be meeting important needs of PwP. Further research using a larger sample size will help to confirm these findings. Care processes were not measured as part of this study which means that inferences cannot be made about the associations between processes and care experiences. In addition, participants were not told to focus on a specific provider and with the potential for participants to see several providers at different settings their experiences can only be considered in general.

## Conclusion

This sample of participants with PD living in Australia reported poor experiences with key aspects of their health care. Whilst further studies are needed, findings from this preliminary study can assist Australian health providers to better target their practice models to deliver quality care for PwP that is patient-centered.

## Electronic supplementary material

Below is the link to the electronic supplementary material.

Supplementary Table 1

## Data Availability

The data that support the findings of this study are available from the corresponding author upon reasonable request and with permission of the Monash Health Human Research Ethics Committee Monash Health Research Services, Clayton, Australia.

## Abbreviations

HRQOL: Health related quality of life
HY: Hoehn & Yahr stage
MDS-UPDRS: Movement Disorders Society-Unified Parkinson’s disease Rating Scale
NMSQ: Non-Motor Symptom Questionnaire
OPS: Overall patient-centeredness score
PCQ-PD: Patient-Centered Questionnaire for Parkinson’s disease
PD: Parkinson’s disease
PDQ-39: Parkinson’s disease Questionnaire-39
PwP: People with Parkinson’s disease
QIS: Quality improvement score
SES: Sub-scale experience score
SI: Summary index
TAFE: Technical and Further Education
VIF: Variance inflation factor
