# Supplementary material for: Health care experiences of people with Parkinson’s disease in Australia
Source: BMC Geriatr. 2023 Jul 12;23:430. doi: 10.1186/s12877-023-04142-3 (PMC10339637; doi:10.1186/s12877-023-04142-3)
Supplement: Supplementary file 1 — Supplementary Table 1 [file 12877_2023_4142_MOESM1_ESM.docx]

**Supplementary Table 1** The number and percentage of participants, both overall and for each group, who saw any of the health professionals for their PD over a 12 month period

| **Health professional** | **All participants**  **(*n*= 227)** | **Group 1**  **(*n* = 82)** | **Group 2**  **(*n* = 38)** | **Group 3**  **(*n* = 39)** | **Group 4**  **(*n* = 68)** |
| --- | --- | --- | --- | --- | --- |
| Neurologist | 216 (95) | 78 (95) | 36 (95) | 38 (97) | 64 (94) |
| Nursing | 81 (36) | 37 (45) | 4 (11) | 12 (31) | 28 (41) |
| PT | 112 (50) | 66 (81) | 12 (32) | 13 (33) | 21 (31) |
| OT | 70 (31) | 42 (51) | 1 (3) | 12 (32) | 15 (22) |
| SP | 81 (37) | 40 (51) | 6 (16) | 12 (31) | 23 (35) |
| Neuropsych | 20 (9) | 12 (16) | 2 (5) | 4 (11) | 2 (3) |
| SW | 38 (17) | 29 (36) | 0 | 5(13) | 4 (6) |
| GP | 151 (68) | 53 (67) | 23 (61) | 23 (61) | 52 (79) |

All data is expressed as raw numbers (percentage)

*PT*  Physiotherapist; *OT* Occupational therapist; *SP* Speech pathologist; *Neuropsych* Neuropsychologist; *SW* Social worker; *GP* General practitioner
